# When Protection Turns Pathogenic: Dual Compartment Functions of Myeloid YB-1 in Renal IRI

**DOI:** 10.3390/ijms27125239

**Published:** 2026-06-10

**Authors:** Anna Leitz, Yili Chen, Xiyang Liu, Yingying Gao, Jialin Wang, Ina Verena Martin, Rafaela Rawinski, Rafael Kramann, Tammo Ostendorf, Ute Raffetseder

**Affiliations:** 1 Department of Nephrology and Clinical Immunology, RWTH Aachen University, Pauwelsstrasse 30, 52074 Aachen, Germany; aleitz@ukaachen.de (A.L.);; 2 Faculty of Medicine, Institute of Immunology, RWTH Aachen University Hospital, 52074 Aachen, Germany; 3 Department of Nephrology, Zhongshan Hospital, Fudan University, Shanghai 200032, China

**Keywords:** acute kidney injury, ischemia–reperfusion injury, YB-1, myeloid cells, neutrophil extracellular traps, immune complexes, Fc gamma receptor CD16, renal inflammation

## Abstract

Acute kidney injury (AKI) caused by ischemia–reperfusion injury (IRI) involves rapid activation of innate immune responses, in which myeloid-derived immune cells critically shape injury severity. Y-box binding protein 1 (YB-1) regulates pro-inflammatory gene expression intracellularly and can be secreted to function extracellularly, yet how these two compartments jointly influence early IRI pathology remains poorly understood. To dissect the roles of intracellular myeloid *versus* extracellular YB-1, we subjected myeloid-specific *Ybx1* knockout, *Ybx1^fl/fl^ × LysM^cre^*, mice and wild-type (WT) littermates to unilateral renal IRI following administration of either a neutralizing anti-YB-1 antibody or control IgG. Kidney injury, inflammation, immune cell recruitment, neutrophil extracellular trap (NET) formation, antibody localization, and Fcγ receptor expression were assessed by qRT-PCR, histology, immunostaining, Western blotting, and flow cytometry. Myeloid-specific knockout of *Ybx1* markedly reduced renal inflammation, neutrophil infiltration, NET formation, and tubular injury. This protective phenotype was lost when extracellular YB-1 was simultaneously reduced: anti-YB-1 treatment in knockout mice restored pro-inflammatory cytokine expression, increased tubular damage markers such as NGAL and KIM-1, exacerbated neutrophil recruitment and NET formation, and led to luminar accumulation of YB-1/anti-YB-1 immune complexes in tubular cells. Mechanistically, *Ybx1*-deficient myeloid cells exhibited significantly reduced CD16 expression, pointing to impaired Fcγ receptor-mediated phagocytosis as the cause of defective immune complex clearance. In contrast, wild-type mice efficiently cleared extracellular YB-1 complexes and showed no injury aggravation upon antibody treatment. Our findings identify myeloid YB-1 as a central regulator of early inflammatory injury in renal IRI and reveal that its protective depletion becomes pathogenic when extracellular YB-1 is simultaneously neutralized, likely due to unmasked defects in immune complex clearance.

## 1. Introduction

Acute kidney injury (AKI) remains a major contributor to morbidity and mortality, particularly in clinical settings involving hemodynamic instability, systemic inflammation, or cardiothoracic surgery, where up to 40% of patients develop AKI and face markedly increased short-term mortality [1,2,3,4,5,6]. Ischemia–reperfusion injury (IRI) represents a prototypical and clinically relevant form of AKI and is widely used as an experimental model because it recapitulates key features of early injury [1,7]. Interruption of renal blood flow, whether systemic, as in shock or cardiac arrest, or local, as during transplantation or vascular clamping, leads to an imbalance between oxygen supply and metabolic demand, accumulation of toxic metabolites, and subsequent reactive oxygen species (ROS) formation, vascular alterations, leukocyte recruitment, and tubular cell death [7,8,9,10,11].

The inflammatory response is a major determinant of injury severity and repair. Intrarenal immune cells rapidly release cytokines such as interleukin (IL)-1 and IL-6 and chemokines, including CCL5 and CCL2/Monocyte Chemoattractant Protein (MCP)-1, to recruit circulating leukocytes to the site of injury [12,13,14]. Neutrophils are among the earliest responders, guided by chemoattractants such as CXCL1, CXCL2, and Leukotriene (LT)B4, initiating rolling, adhesion, and transendothelial migration [14,15,16,17]. Although essential for pathogen control, neutrophils can aggravate tissue damage through release of proteases, ROS, and neutrophil extracellular traps (NETs) [18,19,20]. Subsequently, monocytes infiltrate the kidney and differentiate into macrophages, which may adopt either pro-inflammatory (M1) or pro-repair (M2) phenotypes, thereby influencing the balance between injury progression and resolution [13,14,17,21]. If inflammation is excessive or unresolved, maladaptive repair can lead to fibrosis and chronic loss of kidney function [22].

Y-box binding protein 1 (YB-1, gene name *Ybx1*) has emerged as a key regulator of renal inflammation. It controls the expression of pro-inflammatory mediators such as IL-6 and CCL5 and modulates monocyte and macrophage responses [8,11,23,24,25,26]. Partial reduction in *Ybx1* expression has been shown to ameliorate AKI by suppressing CCL5 induction [27]. In addition to its intracellular roles, YB-1 can be actively secreted *via* a non-classical, vesicle-dependent pathway [26,27,28]. Once released, extracellular YB-1 promotes inflammation, chemotaxis, and mitogenic signaling and can exacerbate tubular injury in AKI models [23,27]. Although YB-1 has emerged as a key inflammatory regulator, the integration of its intracellular and extracellular functions, as well as its cell type-specific roles during early kidney injury, remains largely unresolved.

A recent study provided a compelling therapeutic perspective: neutralizing extracellular YB-1 significantly reduced tubular injury and NET formation in murine IRI, and YB-1 was identified as a structural and functional component of the NET complex [23]. Because NET degradation can mitigate IRI-induced AKI, targeting extracellular YB-1 may represent a promising strategy for limiting renal damage [29].

Here, we show for the first time that although myeloid *Ybx1* deficiency is protective in renal IRI, simultaneous neutralization of extracellular YB-1 negates this benefit, likely by exposing an intrinsic impairment in phagocytic clearance.

## 2. Results

### 2.1. Myeloid Ybx1 Deletion Protects Against Renal I/R–Induced Inflammation, but the Effect Is Reversed by Extracellular YB-1 Reduction

**Figure 1:**
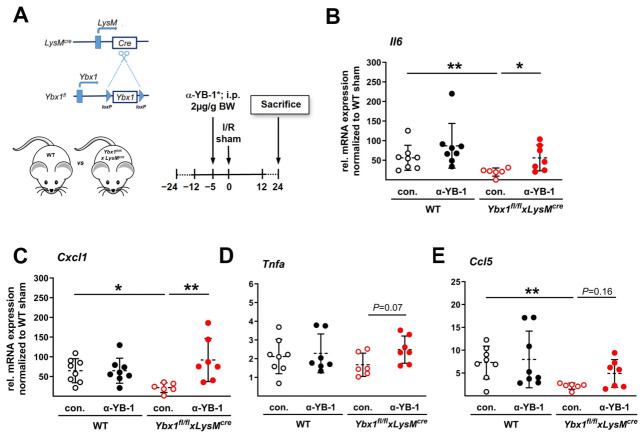

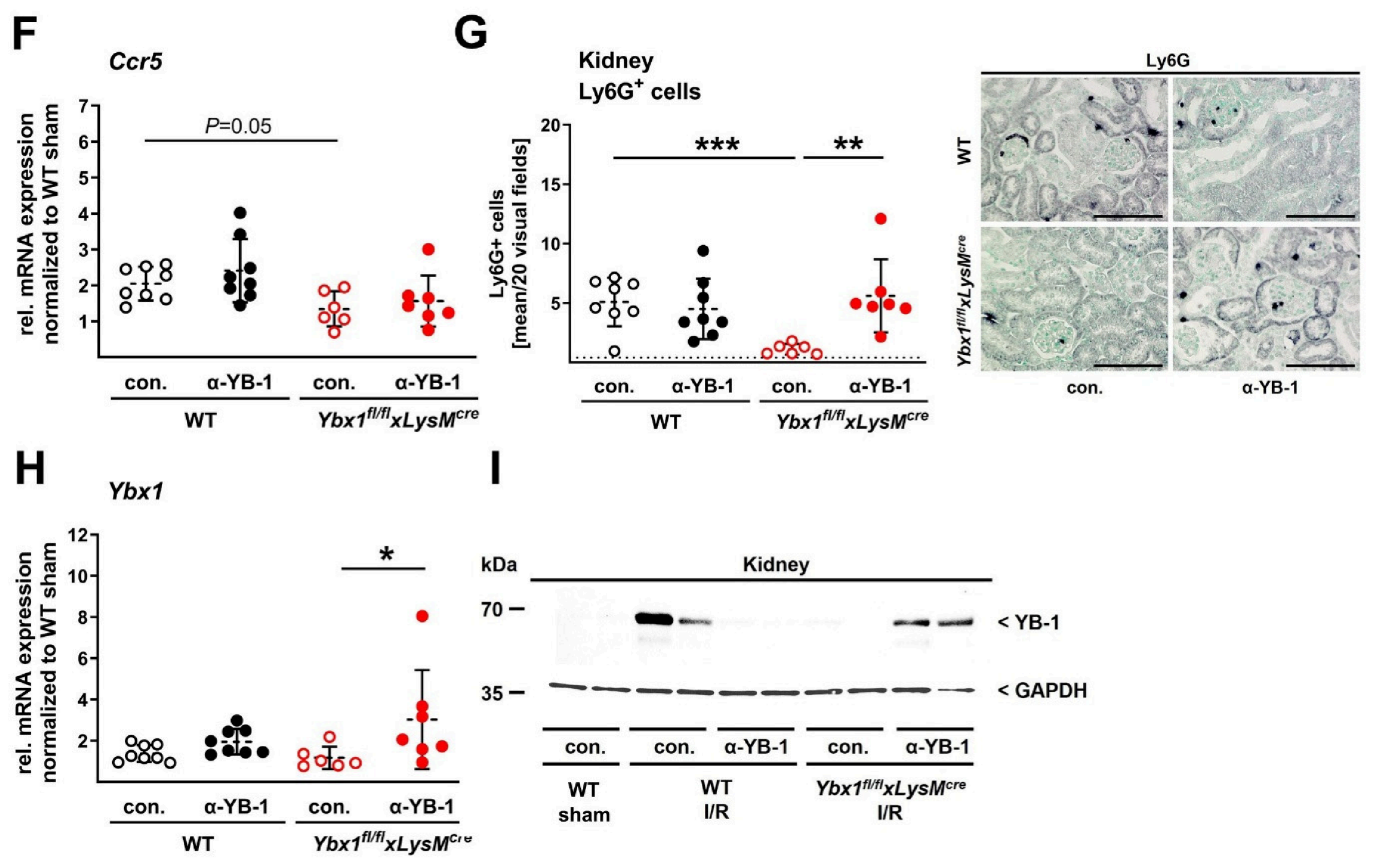
Post-I/R decrease in kidney inflammatory markers in *Ybx1^fl/fl^ × LysM^cre^* mice was reversed by anti-YB-1 (ab) application prior to surgery. (**A**) Myeloid cell-specific *Ybx1*-deficient mice were generated by crossing *Ybx1^fl/fl^* mice with *LysM^Cre^* mice. *Ybx1^fl/fl^ × LysM^cre^* mice and their WT littermates were injected with either anti-YB-1 ab or unspecific IgG (con.) for controls before sham or I/R surgery. The mice were sacrificed 24 h post-surgery. (**B**–**F**) The expression of inflammation-related genes *Il6* (sham WT con. 1.08 ± 0.46) (**B**), *Cxcl1* (sham WT con. 1.31 ± 0.48) (**C**), *Tnfa* (sham WT con. 1.1 ± 0.52) (**D**), *Ccl5* (sham WT con. 1.04 ± 0.32) (**E**), and *Ccr5* (sham WT con. 1.08 ± 0.42) (**F**) was decreased in left kidney tissue mRNA extracts of *Ybx1^fl/fl^ × LysM^cre^* mice injected with IgG control. However, when anti-YB-1 ab was injected, this reduction was reversed. (**G**) Infiltrating Ly6G^+^ neutrophils were decreased in *Ybx1^fl/fl^ × LysM^cre^* mice compared to WT mice injected with unspecific IgG control (con.) (sham WT con. 0.4 ± 0.35). However, injection of anti-YB-1 ab abolished this reduction. Quantification and representative image. The dashed line represents sham-operated WT animals that received an unspecific IgG control. (**H**) Relative *Ybx1* mRNA expression (sham WT con. 1.01 ± 0.11) and (**I**) corresponding YB-1 protein expression in the left kidney of both genotypes after anti-YB-1 ab injection. Gene expression is displayed relative to sham-operated WT animals that received an unspecific IgG control. Sham WT con. (n = 6); IR WT con. (n = 7); IR *Ybx1^fl/fl^ × LysM^cre^* con. (n = 6), IR WT α-YB-1 (n = 8); IR *Ybx1^fl/fl^ × LysM^cre^* α-YB-1 (n = 7); Scale bars = 100 µm; α-YB-1 ab, anti-YB-1 antibody; con., control; GAPDH, glyceraldehyde 3-phosphate dehydrogenase; IL, interleukin; I/R, ischemia–reperfusion; rel., relative; TNF; tumor necrosis factor; WT, wild-type. * *p* < 0.05, ** *p* < 0.01, *** *p* < 0.001.

To dissect the contribution of intra-*versus* extracellular YB-1, *Ybx1^fl/fl^ × LysM^cre^* mice and their wild-type (WT) littermates were subjected to unilateral ischemia–reperfusion (I/R) or sham surgery five hours after administration of either an inhibitory anti-YB-1 antibody or an unspecific rabbit IgG control. Mice were sacrificed 24 h after reperfusion, and kidneys as well as blood samples were analyzed (Figure 1A). To assess baseline effects of anti-YB-1 antibody application, contralateral (non-ischemic) right kidneys were analyzed for the expression of established kidney injury and inflammation markers (*Ngal*, *Kim1*, and *Il6*). No significant differences were observed across genotypes or between control IgG- and anti-YB-1-treatment groups for *Ngal* and *Kim1* expression (Supplementary Figure S1A,B). For *Il6*, only a trend towards reduced expression was observed in the *Ybx1^fl/fl^ × LysM^cre^* mice treated with anti-YB-1, but without statistical significance (Supplementary Figure S1C). Analysis of inflammatory gene expression in I/R kidneys revealed that *Ybx1^fl/fl^ × LysM^cre^* mice displayed a pronounced reduction in the pro-inflammatory mediators *Il6* (Figure 1B), *Cxcl1* (Figure 1C), *Tnfa* (Figure 1D), *Ccl5* (Figure 1E), and its receptor *Ccr5* (Figure 1F) compared with WT animals. In WT mice, anti-YB-1 antibody treatment did not significantly alter cytokine expression relative to IgG controls. In contrast, anti-YB-1 antibody administration to *Ybx1^fl/fl^ × LysM^cre^* mice resulted in a significant upregulation of *Il6* and *Cxcl1*, while *Tnfa*, *Ccl5*, and *Ccr5* only tended to be increased compared with IgG-treated knockout mice. Evaluation of immune cell profiles demonstrated that *Ybx1^fl/fl^ × LysM^cre^* mice exhibited significantly lower circulating neutrophil numbers (Supplementary Figure S2A) and reduced renal infiltration of Ly6G^+^ neutrophils after I/R compared with WT animals (Figure 1G). Anti-YB-1 treatment had no detectable effect in WT mice but caused a marked increase in neutrophil accumulation in the kidney (Figure 1G) and a trend toward elevated circulating neutrophils in knockout mice (Supplementary Figure S2A). Lymphocyte counts were higher in *Ybx1^fl/fl^ × LysM^cre^* mice at baseline, and anti-YB-1 treatment significantly reduced circulating lymphocytes in this group compared with IgG-treated knockouts (Supplementary Figure S2B), consistent with enhanced immune cell recruitment into the injured kidney.

Markers of NET formation further supported a heightened inflammatory response when intracellular myeloid YB-1 was diminished, and extracellular YB-1 was simultaneously targeted (Supplementary Figure S2C). Western blot analysis revealed increased PAD4 expression in WT mice injected with control IgG and in anti-YB-1-treated *Ybx1^fl/fl^ × LysM^cre^* mice, while citrullinated histone H3, a distinct marker of NET formation, was detectable predominantly in *Ybx1^fl/fl^ × LysM^cre^* mice following anti-YB-1 administration (Supplementary Figure S2C, last two lanes).

Analysis of *Ybx1* transcription and YB-1 protein dynamics revealed evidence of compensatory regulation. In knockout mice receiving anti-YB-1 antibody, *Ybx1* mRNA (Figure 1H) and YB-1 protein levels (Figure 1I) were increased, whereas in WT animals the same treatment clearly reduced renal YB-1 protein abundance (Figure 1I). Collectively, these findings demonstrate that myeloid *Ybx1* deletion mitigates renal inflammation after I/R, whereas concomitant targeting of extracellular YB-1 abolishes this protective phenotype and promotes neutrophil recruitment, NET formation, and pro-inflammatory cytokine expression.

### 2.2. Myeloid Ybx1 Deficiency Attenuates Tubular Damage Unless Extracellular YB-1 Is Targeted

**Figure 2:**
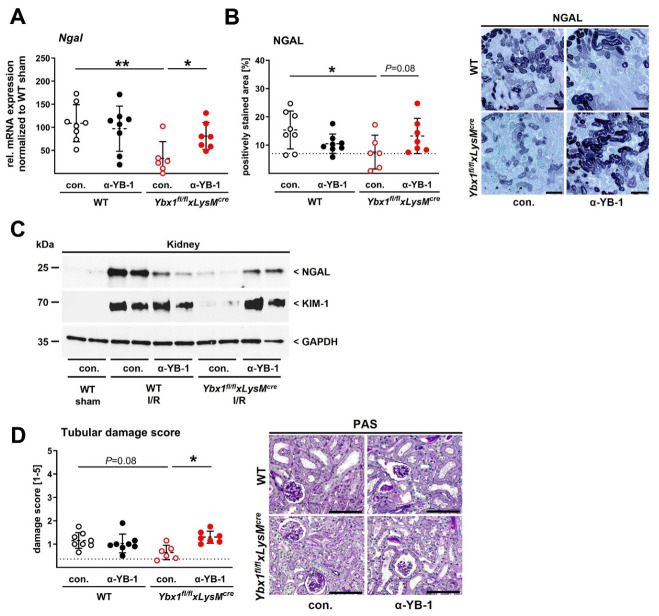
Tubular damage was decreased in *Ybx1^fl/fl^ × LysM^cre^* mice after I/R, which was reversed by anti-YB-1 ab application prior to surgery. (**A**–**C**) Tubular injury marker NGAL was reduced in *Ybx1^fl^^/fl^ × LysM^cre^* compared to WT mice receiving unspecific IgG control (con.). However, this reduction was reversed by the injection of anti-YB-1 antibody. This was evident from the analysis of *Ngal* mRNA (sham WT con. 1.133 ± 0.58) (**A**) and NGAL IHC staining (sham WT con. 7.03 ± 4.16) (**B**). Quantification and representative image are shown. (**C**) Representative immunoblot of NGAL and KIM-1 protein expression and glyceraldehyde 3-phosphate dehydrogenase (GAPDH) as loading control. (**D**) Tubular damage score was lower in *Ybx1^fl/fl^ × LysM^cre^* mice post I/R compared to their WT littermates, which was negated by the application of anti-YB-1 ab (sham WT con. 0.36 ± 0.18). Quantification and representative image are shown. Gene expression is displayed relative to sham-operated WT animals that received an unspecific IgG control. The dashed line represents sham-operated WT animals that received an unspecific IgG control. Sham WT con. (n = 6); IR WT con. (n = 7); IR *Ybx1^fl/fl^ × LysM^cre^* con. (n = 6), IR WT α-YB-1 (n = 8); IR *Ybx1^fl/fl^ × LysM^cre^* α-YB-1 (n = 7); Scale bars = 100 µm; α-YB-1 ab, anti-YB-1 antibody; con, control; I/R, ischemia–reperfusion; KIM, kidney injury molecule; NGAL, neutrophil gelatinase-associated lipocalin; PAS, periodic acid-Schiff; rel., relative; WT, wild-type. * *p* < 0.05, ** *p* < 0.01.

Tubular damage was assessed using the established injury marker NGAL. Gene expression analysis revealed a significant reduction in *Ngal* mRNA levels in *Ybx1^fl/fl^ × LysM^cre^* mice compared with their WT littermates following I/R (Figure 2A). Anti-YB-1 antibody administration did not alter *Ngal* expression in WT mice relative to IgG controls, but significantly increased *Ngal* mRNA levels in *Ybx1^fl/fl^ × LysM^cre^* mice (Figure 2A). Consistent with the transcriptional findings, immunohistochemistry (IHC) staining (Figure 2B), and Western blot analyses (Figure 2C) demonstrated markedly reduced NGAL protein levels in *Ybx1^fl/fl^ × LysM^cre^* mice after I/R compared with WT animals. WT mice tended to exhibit slightly lower NGAL protein levels after anti-YB-1 treatment compared with IgG controls, whereas knockout mice showed increased NGAL levels under these conditions. Similarly, kidney injury molecule-1 (KIM-1) levels were reduced in *Ybx1^fl/fl^ × LysM^cre^* mice compared with WT controls, however this effect was abolished upon anti-YB-1 antibody treatment (Figure 1C). To complement these molecular findings, tubular injury was assessed histologically following Periodic acid-Schiff (PAS) staining of the ischemic left kidney (Figure 2D). Consistent with the reduction in NGAL and KIM-1, the tubular damage score was significantly lower in *Ybx1^fl/fl^ × LysM^cre^* mice following I/R compared with WT mice. Importantly, this protective phenotype was lost after anti-YB-1 antibody injection, which increased tubular injury only in myeloid *Ybx1* knockout animals.

Taken together, these data demonstrate that myeloid *Ybx1* deletion confers substantial protection against tubular damage during renal I/R injury. However, this protective effect is abolished when extracellular YB-1 is simultaneously targeted, resulting in aggravated tubular injury.

### 2.3. Anti-YB-1 Immune Complexes Selectively Accumulate in the Tubular Lumen of Myeloid Ybx1-Deficient Mice

**Figure 3:**
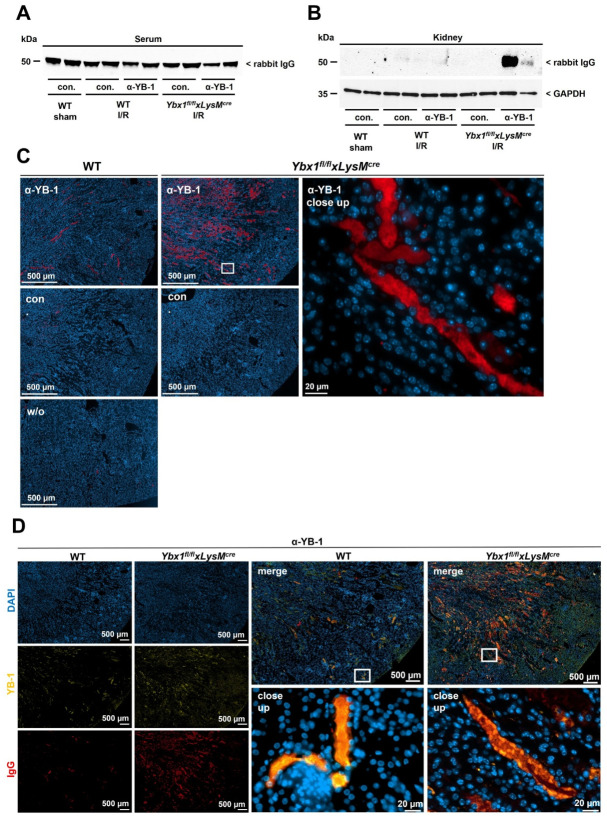
Detection of administered IgG in *Ybx1^fl/fl^ × LysM^cre^* mice and their WT littermates after IR surgery. (**A**,**B**) Representative immunoblots of serum samples (**A**), showing comparable rabbit IgG re-detection across all experimental groups, and of left kidney protein lysates, (**B**), in which rabbit IgG was redetected exclusively in anti-YB-1-treated *Ybx1^fl/fl^ × LysM^cre^* mice. Glyceraldehyde 3-phosphate dehydrogenase (GAPDH) served as a loading control. (**C**) Representative IF stainings for rabbit IgG showed higher amounts of rabbit IgG in the medulla, especially in *Ybx1^fl/fl^ × LysM^cre^* mice injected with anti-YB-1 ab. Rabbit IgG is shown in red and nuclear staining (DAPI) in blue. High-magnification image shows that anti-YB-1 ab accumulated in the tubular lumen. The rectangular area indicates the region shown in the high-magnification image. No rabbit IgG staining was observed in animals injected with unspecific IgG (con.) or without (w/o) antibody injection. (**D**) Representative co-stainings of the administered anti-YB-1 ab (rabbit IgG, red) with YB-1 protein (yellow) demonstrated extensive co-localization, with nearly all anti-YB-1 ab signals overlapping with YB-1. High-magnification images confirmed this finding. α-YB-1 ab, anti-YB-1 antibody; con., control; DAPI, 4′,6-diamidino-2-phenylindole; I/R, ischemia–reperfusion; WT, wild-type; w/o, without.

The fate of the injected inhibitory anti-YB-1 antibody and the unspecific IgG control were monitored after IRI in serum and kidney lysates of WT and *Ybx1^fl/fl^ × LysM^cre^* mice using an anti-rabbit IgG antibody. As shown in Figure 3A, serum antibody levels were comparable across all treatment groups, indicating similar systemic availability. In contrast, anti-YB-1 antibody was detected exclusively in kidney lysates of *Ybx1^fl/fl^ × LysM^cre^*, suggesting impaired clearance of antibody–antigen complexes in the absence of myeloid YB-1 (Figure 3B).

Immunofluorescence staining supported these findings and revealed a markedly stronger anti-YB-1 signal compared with the IgG control, which was again predominantly localized to the medulla of the knockout animals (Figure 3C). WT mice subjected to IRI without antibody injection exhibited no detectable staining (Figure 3C, at the very bottom), confirming the specificity of the observed signal. Higher magnification further demonstrated that antibody accumulation occurred mainly within the tubular lumen (Figure 3C, far right). Co-staining with the distal tubule marker Tamm-Horsfall-Protein (THP) verified localization to distal tubular segments (Supplementary Figure S3). The co-localization of rabbit IgG with YB-1 protein (Figure 3D) confirmed that YB-1/anti-YB-1 immune complexes accumulate in the tubular lumen, and this is markedly more pronounced in *Ybx1^fl/fl^ × LysM^cre^* compared to WT mice.

### 2.4. Myeloid Ybx1 Deficiency Decreases CD16 Expression and Compromises Antibody-Dependent Clearance

**Figure 4:**
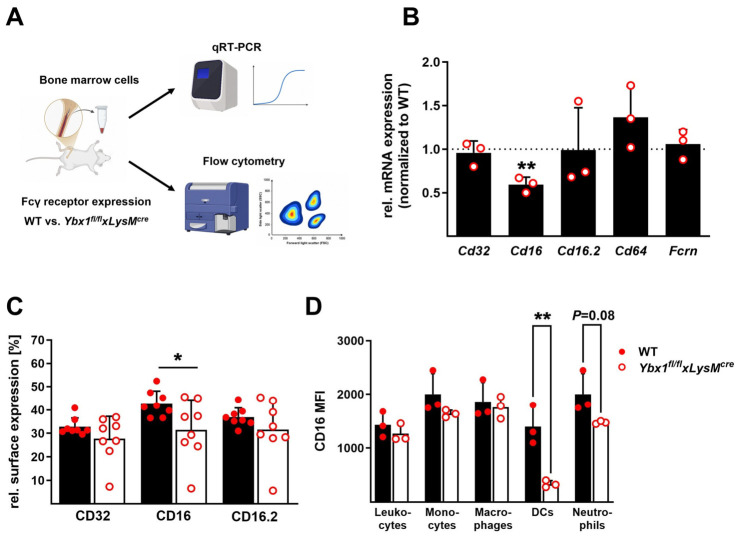
*Ybx1^fl/fl^ × LysM^cre^* mice express lower levels of CD16 receptor in dendritic cells and neutrophils. (**A**) The gene expression levels and surface expression of Fcγ receptors in murine bone marrow cells of *Ybx1^fl/fl^ × LysM^cre^* and WT mice was measured via qRT-PCR or flow cytometry respectively. This figure was created in BioRender. (**B**) Expression of *Cd32* (WT 1.02 ± 0.23), *Cd16* (WT 1.00 ± 0.08), *Cd16.2* (WT 1.18 ± 0.84), *Cd64* (WT 1.03 ± 0.26), and *Fcrn* (WT 1.02 ± 0.23) of all bone marrow cells. Expression levels in bone marrow from *Ybx1^fl/fl^ × LysM^cre^* mice are shown relative to those in WT cells, which are indicated by the dashed line. WT (n = 3); *Ybx1^fl/fl^ × LysM^cre^* (n = 3). (**C**) Cell surface expression of CD32, CD16 and CD16.2 of all bone marrow cells. WT (n = 8); *Ybx1^fl/fl^ × LysM^cre^* (n = 8). (**D**) CD16 MFI in monocytes, macrophages, dendritic cells, and neutrophils of *Ybx1^fl/fl^ × LysM^cre^* compared to WT mice. WT (n = 3); *Ybx1^fl/fl^ × LysM^cre^* (n = 3). CD, cluster of differentiation; DC, dendritic cell; MFI, mean fluorescence intensity; FcRn, neonatal Fc receptor; rel., relative. * *p* < 0.05, ** *p* < 0.01.

Given the diminished clearance of antibody–antigen complexes from the kidneys of *Ybx1^fl/fl^ × LysM^cre^* mice compared with their WT littermates, we hypothesized that these animals may exhibit defects in clearance mechanisms such as phagocytosis. Previous work demonstrated that macrophages lacking YB-1 show impaired phagocytic capacity [9], raising the possibility that antibody-mediated phagocytosis is reduced in this setting and thereby limits immune complex removal, ultimately exacerbating inflammation. To investigate this, the expression of Fcγ receptors, central mediators of antibody-dependent phagocytosis, was examined at both the transcript and protein levels (Figure 4A). Expression analysis of Fc receptor-related genes revealed no significant differences in *Fcrn*, *Cd16.2* or *Cd32* expression between groups, whereas *Cd64* tended to be increased (Figure 4B). In contrast, *Cd16* expression was significantly reduced in bone marrow cells of *Ybx1^fl/fl^ × LysM^cre^* mice compared with WT controls (Figure 4B). Surface expression of the Fcγ receptors CD32, CD16, and CD16.2 was additionally, assessed by flow cytometry, whereas CD64 was excluded due to insufficient signal detection (Figure 4C). Cells were gated based on FSC/SSC characteristics, singlets, and viability before receptor expression was analyzed (Supplementary Figure S4). Consistent with the transcriptional findings, CD16 surface expression was markedly reduced in bone marrow cells from knockout mice (Figure 4C).

To identify the specific myeloid subsets affected, additional flow cytometry analyses were performed using lineage markers for leukocytes (CD45^+^), monocytes (CD45^+^ Ly6C^+^ CD11b^+^), macrophages (CD45^+^ F4/80^+^), dendritic cells (DCs) (CD45^+^ CD11c^+^), and neutrophils (CD45^+^ Ly6G^+^ CD11b^+^) together with CD16 (Supplementary Figure S4; Figure 4D). Mean fluorescence intensity (MFI) measurements confirmed significantly reduced CD16 expression in dendritic cells and a similar trend toward reduced expression in neutrophils of *Ybx1^fl/fl^ × LysM^cre^* mice relative to WT animals.

Taken together, these findings demonstrate that myeloid *Ybx1* deletion compromises renal clearance of anti-YB-1/YB-1 immune complexes, likely due to diminished Fcγ-dependent phagocytic capacity, resulting in luminal immune complex accumulation and ultimately exacerbating kidney injury.

## 3. Discussion

Among the various causes of AKI, IRI remains one of the most clinically relevant, particularly in the settings of renal transplantation and cardiac surgery [30]. Experimental IRI models therefore provide a powerful platform to identify molecular drivers of tissue damage and repair. YB-1 has emerged as a multifunctional regulator of inflammation and cellular stress responses [11,23,27]. In the present study, myeloid-specific deletion of *Ybx1* conferred significant protection against kidney inflammation and tubular injury following IRI. This aligns with previous work showing that whole-body half maximal reduction in *Ybx1* dampens immune responses in acute and chronic inflammation models [27], and supports the conclusion that myeloid YB-1 is a critical mediator of early inflammatory injury during IRI.

Mechanistically, YB-1 activation in immune cells relies on Akt-mediated phosphorylation at serine 102, enabling nuclear translocation and promoter binding [24,31]. Mutations at this site impair nuclear trafficking and abolish induction of inflammatory target genes such as *Ccl5* [32,33]. Consistent with this, *Ybx1^fl/fl^ × LysM^cre^* mice showed robust downregulation of inflammatory mediators, including *Ccl5*, following I/R. Previous studies further demonstrated that neutrophils from WT mice exhibit stronger chemotactic responses toward CCL5 than those from *Ybx1*-deficient animals [27], suggesting that YB-1 supports chemotaxis and thereby neutrophil-driven inflammation. In addition, YB-1 is known to enhance NF-κB signaling, promoting monocyte and macrophage activation [34]. It was reported that *Ybx1^fl/fl^ × LysM^cre^* mice display a shift toward M2 macrophage polarization, a phenotype considered protective during the early phase of IRI when excessive M1 activation exacerbates tissue destruction [35]. Together, these findings indicate that myeloid YB-1 amplifies kidney injury during IRI by promoting pro-inflammatory gene expression, chemotaxis, NF-κB activation, and M1 polarization.

The protective phenotype conferred by myeloid *Ybx1* deletion was abolished when extracellular YB-1 was targeted by anti-YB-1 antibody administration prior to IRI. *Ybx1^fl/fl^ × LysM^cre^* mice treated with anti-YB-1 consistently exhibited increased inflammation in the ischemic kidney and tubular injury compared with knockout mice receiving IgG, a finding that was highly reproducible across all measured parameters. The differential regulation of YB-1 at the mRNA and protein level likely reflects a combination of antibody-mediated effects and secondary inflammatory responses. In WT mice, anti-YB-1 treatment may reduce detectable YB-1 protein through sequestration into immune complexes, followed by enhanced clearance and/or epitope masking. In contrast, in *Ybx1^fl/fl^ × LysM^cre^* mice, where myeloid-derived YB-1 is absent, additional neutralization of extracellular YB-1 may trigger a compensatory increase in *Ybx1* transcription. This response may further be amplified by the enhanced inflammatory milieu observed under these conditions, as YB-1 itself is regulated by inflammatory signaling pathways. Together, these findings highlight that YB-1 does not act as uniformly ‘protective’ or ‘injurious’ but instead exerts context- and compartment-dependent functions, with intracellular YB-1 driving inflammation and extracellular YB-1 forming a distinct pathogenic pool when trapped in immune complexes.

Impaired phagocytic capacity provides a mechanistic basis for this observation. *Ybx1*-deficient macrophages have previously been shown to exhibit defective phagocytosis [35]. In line with this, *Ybx1^fl/fl^ × LysM^cre^* mice displayed reduced expression of Fcγ receptor CD16 in dendritic cells and neutrophils. CD16 is essential for binding and internalization of IgG-containing immune complexes [35,36,37,38] and is expressed on key phagocytic populations, including macrophages, neutrophils, and DCs [39]. Notably, murine Fcγ receptors can bind rabbit IgG [40], the species origin of the anti-YB-1 antibody used here. Reduced CD16 expression therefore likely limits phagocyte-mediated clearance of YB-1/anti-YB-1 immune complexes in knockout mice. As a consequence, higher levels of circulating immune complexes may accumulate in the kidney, where they can amplify inflammation and exacerbate tubular injury [41,42,43]. A similar link between defective immune complex handling and renal injury was recently reported in visceral leishmaniasis, where persistent circulating complexes drive kidney damage due to impaired clearance mechanisms [44], reinforcing the biological relevance of our findings. In addition, our findings are consistent with recent evidence demonstrating that Fcγ receptor IIIA (FCGR3A), the human ortholog of CD16, is highly expressed in monocytes/macrophage populations enriched in inflammatory regions of rejecting kidneys [45]. These cells are thought to respond to IgG-containing immune complexes and contribute to tissue inflammation, supporting the concept that anti-YB-1-induced immune complexes may promote kidney inflammation *via* Fcγ receptor-mediated signaling pathways. Consistent with this interpretation, anti-YB-1 antibody was readily detected in kidneys of *Ybx1^fl/fl^ × LysM^cre^* mice, but only minimally in WT animals or IgG-treated controls. Immunofluorescence revealed pronounced luminal accumulation of antibody signal, predominantly in distal tubules. This distribution can be explained by segmental differences in endocytic capacity: proximal tubules express high levels of megalin and cubilin and efficiently reabsorb filtered proteins and small immune complexes, whereas distal segments lack comparable receptor-mediated uptake mechanisms [46,47], favoring luminal persistence. Interestingly, whole-body *Ybx1* knockout animals exhibit increased megalin expression [48], suggesting a potential YB-1 mechanism that may counteract impaired phagocytic clearance of immune complexes in the absence of YB-1. Co-localization of anti-YB-1 antibody and YB-1 protein further supports the conclusion that YB-1/anti-YB-1 immune complexes become trapped within the tubular lumen, where their persistence likely reinforces injury and further impairs renal clearance [41,42,43]. In addition, impaired phagocytosis has been linked to compensatory increases in NET formation [49]. This relationship is consistent with the enhanced NET-associated markers observed in *Ybx1^fl/fl^ × LysM^cre^* mice treated with anti-YB-1 antibody. 

Together, these findings support a model in which defective immune complex clearance in the absence of myeloid YB-1 results in renal accumulation of extracellular YB-1/antibody complexes, excessive tubular injury, and NET-driven amplification of inflammation. In WT mice, intact phagocytic function prevents this cascade, allowing efficient elimination of immune complexes following anti-YB-1 administration. From a therapeutic perspective, these data suggest that targeting extracellular YB-1 may only be beneficial in settings with preserved phagocytic function, whereas in states of impaired myeloid clearance such intervention could be ineffective or even deleterious.

Differences between the present study and our previous work [23], in which anti-YB-1 treatment more clearly conferred beneficial effects in a bilateral IRI model, likely reflect model-specific differences in injury severity. Bilateral IRI causes more severe systemic injury and may provide a broader window for antibody-mediated protection. By contrast, unilateral IRI, used here to avoid excessive mortality due to unknown effects of myeloid *Ybx1* deletion, results in a milder, more localized injury that can mask protective effects in WT animals [50].

Taken together, these findings identify myeloid YB-1 as a key regulator of inflammation and tissue injury during IRI. While it promotes pro-inflammatory signaling, chemotaxis, and M1 activation, it simultaneously supports Fcγ receptor expression and efficient clearance of immune complexes. The molecular mechanisms by which YB-1 regulates CD16 expression remain an important subject for future investigation, as they may reveal new therapeutic opportunities to modulate immune complex handling and inflammation during AKI. Furthermore, our findings raise the possibility that anti-YB-1 antibodies may not only serve as therapeutic agents but could, if present endogenously, contribute to pathogenic immune complex formation and renal injury. Whether such antibodies exist in human AKI or transplant settings remains unknown and represents a compelling direction for future clinical studies.

## 4. Material and Methods

### 4.1. I/R Surgery and Antibody Application

The local review board approved all animal studies in accordance with prevailing guidelines for scientific animal experimentation. Animals were held in cages with a constant temperature and humidity with drinking water and food *ad libitum*. Reducing the YB-1 content in myeloid cells was accomplished by crossbreeding *LysM^cre^* mice with floxed *Ybx1* (*Ybx1^fl/fl^*) mice on a C57BL/6 background, respectively. In this mouse model, a *Ybx1* allele with *loxP*-flanked *exon 3b* encoding part of the cold shock domain enables Cre-mediated excision, which introduces a premature stop codon and disrupts *Ybx1* expression (Figure 1A). Control groups consisted of littermates negative for the Cre allele or *Ybx1^+/+^LysM^cre^* mice, referred to as WT littermates. Both male and female mice were included in the study. As no obvious sex-related differences were observed in the measured outcomes and sex distribution was not balanced across all experimental groups, sex was not included as an independent variable in the statistical analyses, and data from males and females were analyzed together. Anti-YB-1 antibody (Sigma-Aldrich; Y0396, Steinheim, Germany) or normal rabbit IgG (Cell Signaling; 2729, Danvers, MA, USA) was administered intraperitoneally (i.p.) at a concentration of 2 µg/g body weight (BW) 5 h before surgery. Mice underwent unilateral renal I/R or sham surgery under ketamine/xylazine anesthesia (100/20 mg/kg BW). After flank incision, the left kidney was exposed and renal vessels were clamped for 30 min, followed by clamp removal and visual confirmation of reperfusion. Sham-operated mice underwent the identical procedure without vascular clamping. Wounds were closed in two layers. Postoperative analgesia was provided with buprenorphine (0.1 mg/kg BW, subcutaneously (s.c.) every 4 h during daytime; 0.008 mg/mL in drinking water overnight). Initial group sizes were as follows: sham WT control (n = 7), IR WT control (n = 8), IR *Ybx1^fl/fl^ × LysM^cre^* control (n = 9), IR WT α-YB-1 (n = 11) and IR *Ybx1^fl/fl^ × LysM^cre^* α-YB-1 (n = 7). Based on predefined exclusion criteria, including absence of detectable rabbit IgG serum levels (indicating unsuccessful antibody exposure), surgical complications, or pre-existing health abnormalities (e.g., infected appendix or blood in the bladder), a subset of animals was excluded from the final analysis. Final group sizes were: sham WT control (n = 6); IR WT control (n = 7); IR *Ybx1^fl/fl^ × LysM^cre^* control (n = 6), IR WT α-YB-1 (n = 8); IR *Ybx1^fl/fl^ × LysM^cre^* α-YB-1 (n = 7). Mice were randomly assigned to the different experimental groups prior to surgery and treatment. All animals were housed under identical conditions and allowed to acclimatize before experimentation. Kidney samples were processed in parallel to minimize potential confounding effects. Histological scoring and quantitative outcome analyses were performed in a blinded manner, whereas investigators performing surgeries and antibody treatments were aware of group allocation. Renal injury and inflammation were assessed by histological tubular damage scoring as well as by analysis of kidney injury and inflammatory markers using qRT-PCR, immunohistochemistry, and Western blotting. Primary outcome measures were tubular injury and renal inflammatory marker expression following I/R injury.

### 4.2. mRNA Extraction and qRT-PCR

For quantitative gene expression analysis qRT-PCR in kidney tissue RNA extraction, cDNA synthesis and qRT-PCR were performed as described previously [11]. Primer sequences and probes are listed in Supplementary Table S1.

### 4.3. Protein Extraction and Western Blot Analysis

Protein extraction and Western blotting from kidney tissue were performed as described elsewhere [11,27]. Equal loading of serum proteins was ensured by Ponceau S staining. Antibodies used for Western blotting are listed in Supplementary Table S2.

### 4.4. Immunohistochemistry and Immunofluorescence

Immunohistochemical analyses and quantifications for NGAL, Ly6G, and PAS staining were performed as described [51,52,53]. Tubular injury was scored in PAS stainings on a scale of 0–5: 0 = none; 1 = less than 10%; 2 = 11–25%; 3 = 26–50%; 4 = 51–75%; 5 = more than 75% affected tubules. The tubulointerstitial injury was defined as inflammatory cell infiltrates, tubular dilation and/or atrophy, or interstitial fibrosis. The total score was calculated as the average of all tubular scores. Immunofluorescence staining was performed on 1 µm sections of formalin-fixed, paraffin-embedded kidney tissue. After deparaffinization, antigen retrieval was carried out in citrate buffer. Sections were bleached, blocked with donkey serum, and incubated overnight at 4 °C with primary antibodies. Following PBS washes, appropriate secondary antibodies were applied, nuclei were counterstained with 4′,6-diamidino-2-phenylindole (DAPI), and slides were mounted. Fluorescence signals were evaluated qualitatively using a ZEISS LSM 990 microscope (ZEISS, Oberkochen, Germany). The antibodies are listed in Supplementary Table S2.

### 4.5. Isolation of Murine Bone Marrow Cells

Untreated *Ybx1^fl/fl^ × LysM^cre^* mice and their WT littermates were euthanized by cervical dislocation under isoflurane anesthesia. The femur and fibula of the mice were disconnected, and the muscle tissue was removed. The bone was opened using bone scissors, the cells were extracted from the bone by centrifugation at 2000× *g* for 2 min at 4 °C, and the cells were washed three times with PBS.

### 4.6. Flow Cytometry

Murine bone marrow cells were stained with fluorophore-conjugated antibodies against selected surface markers (for antibodies see Supplementary Table S2). Cells were incubated with antibody mixes in PBS for 15 min at 4 °C in the dark, washed once, and analyzed on a BD LSRFortessa™ flow cytometer. Data were processed using FlowJo v11. Gating included forward scatter (FSC)/side scatter (SSC) profiles, singlet discrimination, and exclusion of dead cells using Zombie Aqua™ viability dye (Biolegend; 423101, San Diego, CA, USA). In the gated populations CD16 MFI was calculated.

### 4.7. Statistical Analyses

Data were analyzed using GraphPad Prism10 software. Results are presented as mean ± SD. Student’s *t*-test was used to compare 2 experimental groups; whenever >2 groups were compared, 2-way ANOVA was applied. Data was tested for normality using Shapiro–Wilk test and Kolmogorov–Smirnov test. Potential outliers were identified using the robust regression and outlier removal (ROUT) method (Q = 1%) implemented in GraphPad Prism. Significance levels were * *p* < 0.05, ** *p* < 0.01, *** *p* < 0.001, and **** *p* < 0.0001.

## Data Availability

The original contributions presented in this study are included in the article/Supplementary Material. Further inquiries can be directed to the corresponding author.

## Supplementary Materials

The following supporting information can be downloaded at: https://www.mdpi.com/article/10.3390/ijms27125239/s1.
