# Supplementary material for: When Protection Turns Pathogenic: Dual Compartment Functions of Myeloid YB-1 in Renal IRI
Source: Int J Mol Sci. 2026 Jun 10;27(12):5239. doi: 10.3390/ijms27125239 (PMC13299933; doi:10.3390/ijms27125239)
Supplement: Supplementary file 1 [file ijms-27-05239-s001.zip › ijms-4201923-supplementary.pdf]

Supplementary material

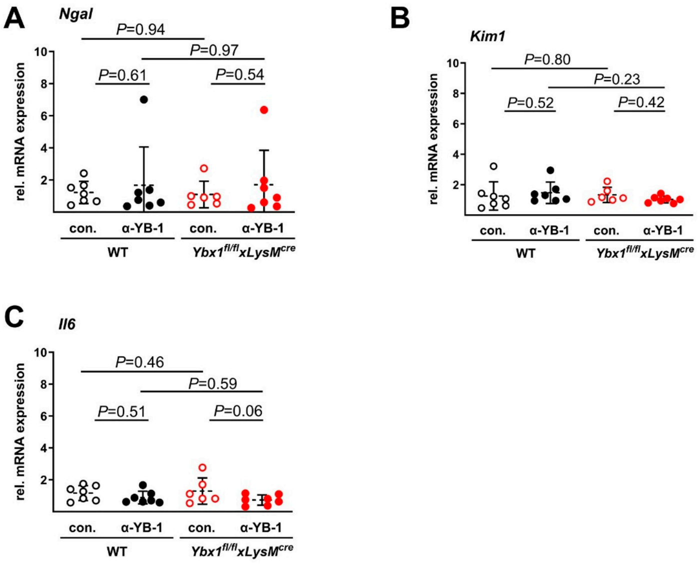

Supplementary Figure S1. Expression of kidney injury and inflammatory markers in contralateral kidneys remains unchanged between genotypes and anti–YB-1 treatment groups.

Contralateral (non-ischemic) kidneys were analyzed for the expression of the kidney injury markers *Ngal* (A) and *Kim1* (B), as well as the inflammatory marker *Il6* (C). No significant differences were observed between genotypes or treatment groups. Sham con. (n=6); IR WT con. (n=8); IR *Ybx1*$^{fl/fl}$×*LysM*^{cre} con. n=6, IR WT α-YB-1 (n=8); IR *Ybx1*$^{fl/fl}$×*LysM*^{cre} α-YB-1 (n=7); α-YB-1 ab, anti-YB-1 antibody; con., control; KIM-1, kidney injury molecule-1; NGAL, neutrophil gelatinase-associated lipocalin; rel., relative; WT, wild type.

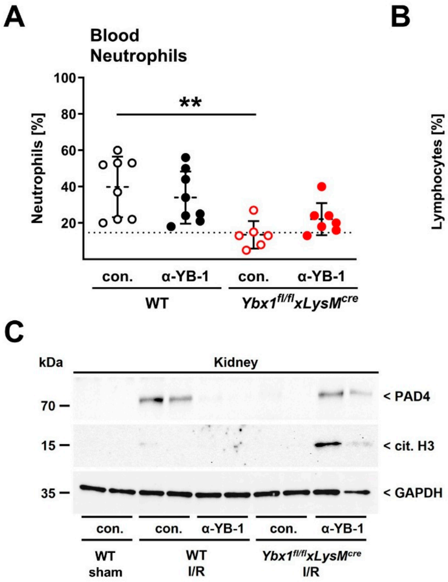

Supplementary Figure S2: Anti-YB-1 treatment alters circulating immune cell profiles and promotes NET formation in myeloid *Ybx1*-deficient mice

A. The percentage of blood neutrophils was decreased in *Ybx1$^{fl/fl}$×LysM^{cre}* mice compared to WT mice injected with unspecific IgG control (con.) (sham WT con. 14.2 % ± 7.9 %). **B.** In *Ybx1$^{fl/fl}$×LysM^{cre}* mice receiving IgG control blood lymphocyte levels where increased compared with WT animals, whereas anti-YB-1 injection significantly reduced lymphocyte numbers exclusively in knock out mice (sham WT con. 74.8 % ± 28.9 %). **C.** Representative immunoblots of the NET component citrullinated H3 and the enzyme PAD4 revealed higher expression in *Ybx1$^{fl/fl}$×LysM^{cre}* mice after anti-YB-1 ab injection. The dashed line represents sham-operated WT animals that received an unspecific IgG control. Sham con. (n=6); IR WT con. (n=8); IR *Ybx1$^{fl/fl}$×LysM^{cre}* con. (n=6), IR WT α-YB-1 (n=8); IR *Ybx1$^{fl/fl}$×LysM^{cre}* α-YB-1 (n=7); α-YB-1 ab, anti-YB-1 antibody; citr. H3; citrullinated histone H3; GAPDH, glyceraldehyde 3-phosphate dehydrogenase; I/R, ischemia reperfusion; NET, neutrophil extracellular trap; PAD, protein arginine deiminase; WT, wild-type. ** $p < 0.01$, *** $p < 0.001$.

Commented [M1]: All special symbols should explain in caption, we added explanations for *, **, *** here, please confirm. Same as below figures.

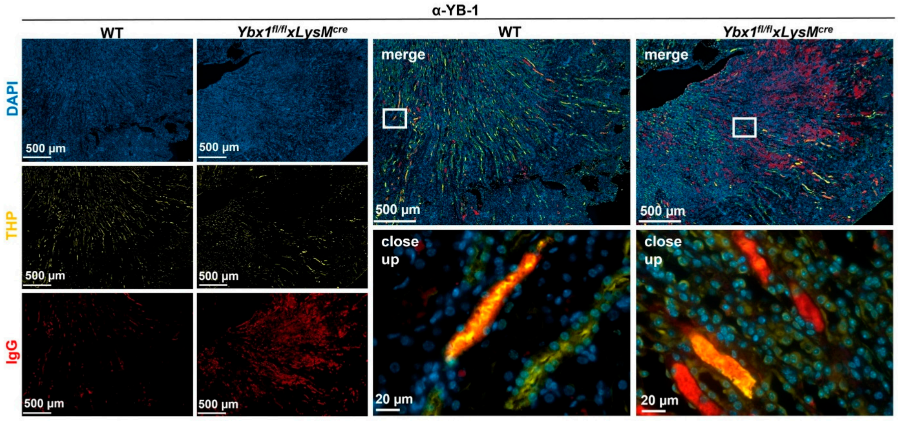

Supplementary Figure S3: Co-localization of administered anti-YB-1 ab and distal tubule marker THP in *Ybx1$^{fl/fl}$*×*LysM^{cre}* mice and their WT littermates after IR surgery

Representative co-staining of the administered anti-YB-1 ab (rabbit IgG) with the distal tubule marker THP reveals a strong accumulation of anti-YB-1 ab within distal tubules. High-magnification images further confirm this co-localization. Nuclei (4',6-diamidino-2-phenylindole (DAPI)) are shown in blue, THP in yellow, and rabbit IgG in red. The rectangular area marks the region displayed in the close-up image. DAPI, 4',6-diamidino-2-phenylindole; THP, Tamm-Horsfall protein; WT, wild-type.

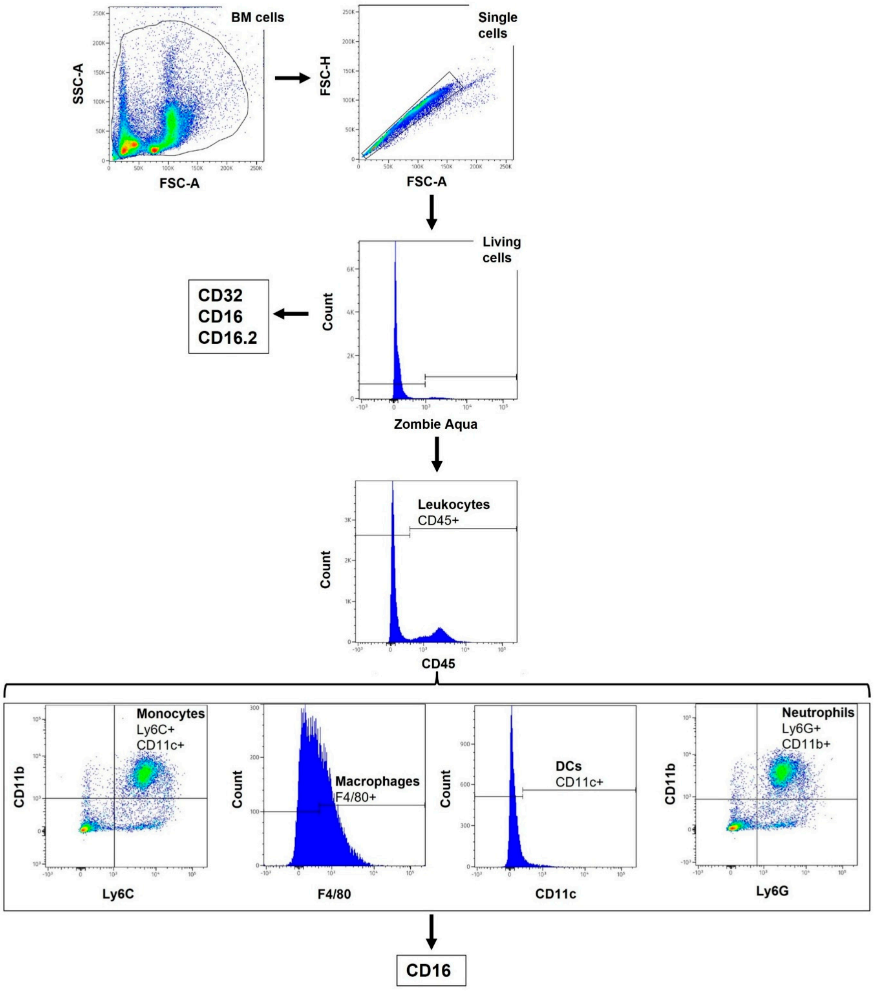

Supplementary Figure S4: Gating strategy for flow cytometry

Bone marrow cells were first gated based on forward scatter (FSC) and side scatter (SSC). Single cells were subsequently identified, followed by exclusion of dead cells using Zombie Aqua™ viability dye. Surface expression of CD32, CD16, and CD16.2 was then analyzed, or CD16 mean fluorescence intensity (MFI) was determined in the indicated myeloid cell subsets CD; cluster of differentiation; DC, dendritic cell; FSC-A, forward scatter area; FSC-H, forward scatter height; SSC-A, side scatter area

Supplementary Table S1: Primers and probes for qRT-PCR

Gene	Forward Primer	Reverse Primer
Ccl5	5'-AGT GCT CCA ATC TTG CAG TCG-3'	5'-CAC TTC TTC TCT GGG TTG GCA-3'
Cd64	5'-AGG TTC CTC AAT GCC AAG TG-3'	5'-ATT CTT CCA TCC GTG ACA CC-3'
Cd32	5'-CTA GGA AGG ACA CTG CAC CA-3'	5'-GAC AGC AAT CCC AGT GAC AG-3'
Cd16	5'-TAT CGG TGT CAA ATG GAG CA-3'	5'-TAT GGC ACC TTA GCG TGA TG-3'
Cd16.2	5'-TCC GGA TAT CTG TGG TGA CA-3'	5'-GCT TGA ATG CCA GAG AAA GC-3'
Fcrn	5'-ACT GCT AGG CCA CCT GGA G-3'	5'-AGG AGA AAG CAGCAC AGG TC-3'
Gapdh	5'-GGC AAA TTC AAC GGC ACA GT-3'	5'-AGA TGG TGA TGG GCT TCC C-3'
Il6	5'-TGT TCA TAC AAT CAG AAT TGC CAT T-3'	5'-AGT CGG AGG CTT AAT TAC ACA TGT T-3'
Kim1	5'-ACA TAT CGT GGA ATC ACA ACG AC-3'	5'-ACA AGC AGA AGA TGG GCA TTG-3'
Ngal	5'-TGG CCC TGA GTG TCA TGT G-3'	5'-CTC TTG TAG CTC ATA GAT GGT GC-3
Tnfa	5'-CCC TCA CAC TCA GAT CAT CTT CT-3'	5'-GCT ACG ACG TGG GCT ACA G-3'
**Gene**	**Probe**	
18s	Hs99999901 s1	
Ccr5	Mm01216171 m1	
Cxcl1	Mm04207460 m1	
Ybx1	Hs02742754 m1	

Supplementary Table S2: Antibodies used for Western blot, flow cytometry and immuno stainings

Tool	Catalogue Number	Manufacturer
CD11b-Brilliant Violet 650™	101259	BioLegend, San Diego, CA, USA
CD11c-APC/Cyanine7	117324	BioLegend, San Diego, CA, USA
CD16-PE/Cyanine7	158015	BioLegend, San Diego, CA, USA
CD16.2-PE	149503	BioLegend, San Diego, CA, USA
CD32-FITC	156407	BioLegend, San Diego, CA, USA
CD45-PerCP/Cyanine5.5	103132	BioLegend, San Diego, CA, USA
F4/80-APC	17-4801-82	eBioscience, San Diego, CA, USA
GAPDH	NB 300-221	Novus Biologicals, Wiesbaden-Norderstadt, Germany
Histone H3 (citrulline R2, R8, R17)	ab5103	Abcam, Cambridge, UK
Kim-1	AP08825PU-N	Acris, Heidelberg, Germany
Ly6C-PE	560592	BD Biosciences, Heidelberg, Germany
Ly6G		BD Biosciences Heidelberg, Germany
Ly6G-Brilliant Violet 711™	127643	BioLegend, San Diego, CA, USA
NGAL	AF1857	R&D Systems, Wiesbaden-Norderstadt, Germany
PAD4	GTX113945	GeneTex, Irvine, CA, USA
THP	MAB5175	Biotechne, Wiesbaden-Norderstadt, Germany
YB-1$^{C\text{-}term.}$	Y0396	Sigma-Aldrich, Steinheim, Germany

YB-1	GTX113945	BioXCell, Teisendorf, Germany
Goat IgG-HRP	P 0160	Dako, Hamburg, Germany
Goat IgG H&L Biotin	BA 5000	Vector Laboratories, Newark, CA, USA
Mouse IgG H&L HRP	P 0160	Dianova, Hamburg, Germnay
Mouse IgG Alexa-555	A21127	Life Technologies, Darmstadt, Germany
Rabbit IgG H&L HRP	P 0448	Dako, Hamburg, Germany
Rabbit IgG Alexa-647	A 31573	Invitrogen, Waltham, MA, USA
Rat IgG H&L Biotin	BA 4001	Vector Laboratories, Newark, CA, USA
Rat IgG DyLight®-550	SAS 10027	Invitrogen, Waltham, MA, USA
